# Global latitudinal patterns in leaf herbivory are related to variation in climate, rather than phytochemicals or mycorrhizal types

**DOI:** 10.1093/nsr/nwad236

**Published:** 2023-09-08

**Authors:** Hui Tang, Xianhui Zhu, Yonglin Zhong, Yuanzhi Li, Wenqi Luo, Hanlun Liu, Patrice Descombes, Alan C Gange, Chengjin Chu

**Affiliations:** State Key Laboratory of Biocontrol, School of Ecology/School of Life Sciences, Sun Yat-sen University, China; State Key Laboratory of Biocontrol, School of Ecology/School of Life Sciences, Sun Yat-sen University, China; Guangdong Provincial Key Laboratory of Silviculture, Protection and Utilization, Guangdong Academy of Forestry, China; State Key Laboratory of Biocontrol, School of Ecology/School of Life Sciences, Sun Yat-sen University, China; State Key Laboratory of Biocontrol, School of Ecology/School of Life Sciences, Sun Yat-sen University, China; State Key Laboratory of Biocontrol, School of Ecology/School of Life Sciences, Sun Yat-sen University, China; Musée et Jardins botaniques cantonaux, Switzerland; Department of Ecology and Evolution, University of Lausanne, Switzerland; Department of Biological Sciences, Royal Holloway University of London, UK; State Key Laboratory of Biocontrol, School of Ecology/School of Life Sciences, Sun Yat-sen University, China

Plant-herbivore interactions are among the most important factors shaping plant community dynamics and ecosystem functioning. The latitudinal herbivory hypothesis proposed that insect herbivory rates decline with increasing latitudes owing to benign environmental conditions in the tropics, but studies have produced contradictory conclusions [1–3]. The sources of this controversy have been suggested to result from variation in sampling methodologies, study systems, climatic/biogeographic zones and types of herbivore studied by different authors [2,3]. Resolving this controversy should encourage more studies to explore specific underlying mechanisms through novel insights into the complex abiotic (such as climate) and biotic factors (such as phytochemical diversity and plant mycorrhizal types) that influence levels of plant attack by invertebrates.

Climate might be pivotal in driving latitudinal patterns in herbivory by impacting the abundance and activity of herbivores, as well as the physiological and defensive traits of host plants. The intertwined latitude-climate-herbivory relationships depict the way that climatic variables change with latitudes and the way that climatic variables affect herbivory. Traditionally, most studies focused on MAT (mean annual temperature) and MAP (mean annual precipitation) but ignored other potentially important climatic factors [2,3], such that the true role of climate in driving geographic variations in herbivory may be masked.

Theory often assumes greater herbivore pressure towards tropical regions where host plants with higher defense levels should be selected for [1,2]. However, latitudinal variation in herbivory could also be the consequence, rather than the cause, of opposing latitudinal trends of plant anti-herbivore defenses. Phytochemical compounds provide effective anti-herbivore defense, but there is typically no ‘common currency’ for measuring defensive phytochemicals for all species in a community, despite the relatively weak evolutionary constraints on the evolution of phytochemicals at the plant family level [4]. Phytochemical diversity (e.g. richness and evenness of chemical compounds) across species may reflect the net chemical defense of plants. A mixture of multiple phytochemicals may act synergistically, additively or antagonistically to influence herbivore performance, but we still have little knowledge on how compounds from different chemical classes would interact to determine the levels of herbivory [5]. Together with its variation along environmental gradients, phytochemical diversity may ultimately influence the global trends of herbivory rates, but this idea remains untested. Furthermore, mycorrhizal symbioses have the potential to systemically induce changes in the whole plant metabolome and/or plant nutrition to influence aboveground leaf herbivory [6,7]. Thus, incorporating a third trophic level, i.e. mycorrhizal fungi in plant-herbivore interactions can potentially contribute to explain the latitudinal variation in herbivory patterns. However, different mycorrhizal types may vary in magnitude and direction of effects on plant-herbivore interactions due to inherent differences in mycorrhizal traits [8,9]. Moreover, different plant mycorrhizal types display discordant latitudinal distributions: the relative proportion of arbuscular mycorrhizal (AM) plants nonlinearly declines towards the poles, while ectomycorrhizal (EcM) plants and nonmycorrhizal (NM) plants show reversed latitudinal trends [10]. Our novel approach is to integrate this biogeographical pattern of mycorrhizae with potential mycorrhizal-mediated variation in herbivory, within the framework of the latitudinal herbivory hypothesis [1–3].

To reveal global latitudinal patterns in herbivory and the underlying determinants, we compiled a large dataset of herbivory rates, abiotic factors (temperature and precipitation) and biotic factors (phytochemical diversity and plant mycorrhizal types) from published materials and conducted a quantitative synthesis by building upon earlier studies [4,11,12]. In total, our dataset included 2702 data points for 1562 seed plant species belonging to 172 families. Among the herbivory data, 1504 data points at 494 locations from 268 studies were compiled in Zhang *et al.* [11], 222 data points at 57 locations in Mendes *et al.* [12] and the remaining 976 data points at 340 locations from 96 studies searched by ourselves. The average leaf herbivory level was 6.92% (SE = 0.167%, *N* = 2702) across all plants. We first performed phylogenetic generalized linear mixed model (PGLMM) analyses to explore the latitude-herbivory relationship while accounting for the phylogenetic relationship among plant species involved. We then conducted similar PGLMM analyses to test the effects of several climatic variables and their quadratic terms along with phytochemical diversity, plant growth form and mycorrhizal type on herbivory. Finally, we analyzed latitudinal variations of these climatic variables, phytochemical diversity and plant mycorrhizal type to understand how they influence global latitudinal patterns in herbivory.

We found a hump-shaped relationship between latitude and herbivory at the global scale when elevation was included as a covariate in the model (Fig. 1B; Supplementary Table S1). These results were confirmed when we used elevation-adjusted latitudes in model analyses by converting the magnitude of upward shift in elevations to that of poleward shift in latitudes (Supplementary Fig. S1B). Furthermore, random resampling analyses also supported quadratic over linear models and showed statistical significance of the quadratic latitude term (Supplementary Fig. S1C, D). It was notable that low-elevation tropical regions had relatively low levels of herbivory (Supplementary Fig. S1A), which contributed to a reduction in the average herbivory level in the tropics.

**Figure 1.**
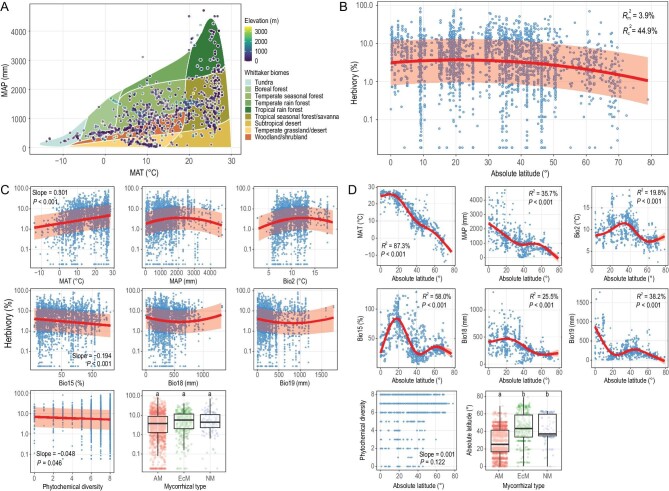
The global latitudinal pattern in leaf herbivory and the potential drivers. (A) Distribution of 890 study locations in different biomes. (B) The global latitudinal pattern in leaf herbivory when accounting for the elevation effect. The tick labels of the y axis were log_10_-transformed to better visualize herbivory rates at their original scale. Shown are the marginal R-squared value ($R_{\mathrm{m}}^2$) which represents the proportion of the variance explained by fixed effects, and the conditional R-squared value ($R_{\mathrm{c}}^2$) which represents the proportion of the variation explained by both fixed and random effects. The red curve and shadow indicated the fitted line and its 95% confidence interval, respectively. (C) The marginal effect of each predictor variable on leaf herbivory when accounting for other variables in the PGLMM model. Variable abbreviations: MAT, mean annual precipitation; MAP, mean annual precipitation; Bio2, mean diurnal range; Bio15, precipitation seasonality; Bio18, precipitation of warmest quarter; Bio19, precipitation of coldest quarter. Plant mycorrhizal type: arbuscular mycorrhizal (AM), ectomycorrhizal (EcM), nonmycorrhizal (NM). Letters above the boxplots indicate groups identified by Tukey's HSD multiple comparison analyses. (D) The latitudinal distribution of climates, phytochemical diversity and plant mycorrhizal type. Climate-latitude relationships were fitted based on generalized additive models (GAM).

We found that two temperature-related variables and four precipitation-related variables had significant effects on herbivory and its latitudinal pattern (Fig. 1C, Supplementary Fig. S3; Supplementary Tables S3, S4). MAT intensified herbivory and generally decreased with latitudes except in the tropics where MAT was relatively stable, which contributed to a less striking herbivory pattern in the tropics but a decreasing latitudinal pattern outside the tropics. High levels of MAP in the tropics reduced herbivory in those areas. However, moderate Bio2 (mean diurnal range), high Bio18 (precipitation of warmest quarter) and Bio19 (precipitation of coldest quarter) in the tropics could increase herbivory in this region. Bio15 (precipitation seasonality) reduced herbivory and decreased with increasing latitudes in the tropics, which could have contributed to a decreasing latitudinal herbivory pattern in this region. Together, MAT and MAP partially counteracted the effects of those other four climatic variables above, which finally led to a hump-shaped latitudinal herbivory pattern. Therefore, our results only partly supported the latitudinal herbivory hypothesis, that is, we only found a negative latitude-herbivory relationship in temperate and polar regions but not in tropical regions.

In our dataset, most plants were sampled from families that contain six or more broad classes of phytochemicals (Supplementary Fig. S4). We showed that plants from families with more classes of phytochemicals only weakly tended to have a lower level of herbivory (Fig. 1C, Supplementary Fig. S3; Supplementary Tables S3, S4), so we did not find convincing evidence of synergistical, additive or antagonistic interactions among phytochemicals from eight different classes. Additionally, phytochemical diversity was independent of latitudes (Fig. 1D). These results suggest that family-level data of phytochemical diversity failed to help explain the global latitudinal patterns in herbivory. It is worth noting that more extensively studied families tended to have more classes of phytochemicals (Supplementary Fig. S5), but there was no such positive correlation for plant families that have more than three classes of phytochemicals (Supplementary Fig. S5), indicating that plant families found to contain very few classes of phytochemicals have so far been underexplored [4].

Plant mycorrhizal type had no statistically significant effects on herbivory. AM plants, EcM plants and NM plants all had similar herbivory levels, with an average herbivory rate (mean ± SE) of 6.53% ± 0.201%, 7.78% ± 0.397% and 8.27% ± 1.020%, respectively (Fig. 1C; Supplementary Tables S3, S4). This conclusion remained when accounting for the potential confounding effect of plant growth form, given that EcM plants are mostly woody species (Supplementary Fig. S6) [10]. EcM plants may be more resistant to soil pathogens than AM and NM plants [8], but we did not find a similar pattern for leaf herbivory. AM plants generally dominated in lower latitudes, while EcM plants and NM plants were primarily distributed in higher latitudes (χ^2^ = 270.31, *df* = 2, *P* < 0.001; Fig. 1D). Taken together, the biogeographic pattern of mycorrhizal types seemed not to influence the latitudinal patterns in herbivory. Moreover, woody plants only suffered marginally significantly higher herbivory than non-woody plants, with an average herbivory rate of 7.69% ± 0.202% versus 5.54% ± 0.288%, respectively (Supplementary Tables S3, S4), which showed limited evidence for the plant apparency theory [11].

In summary, a hump-shaped latitudinal pattern in leaf herbivory was observed based on currently available data, inconsistent with the latitudinal herbivory hypothesis. It is climate, rather than phytochemical diversity or plant mycorrhizal types, that mostly shapes the global pattern of plant-herbivore interactions. Importantly, latitudinal variations in temperature and precipitation can limit the diversity, abundance and activity of herbivores and thus herbivory intensity [13]. The limited power of phytochemical data at the plant family level here pointed to a more straightforward and effective way of sampling detailed species-level phytochemical data along latitudes in future studies, which could help to address the unexplained variation in herbivory. Mycorrhizal-mediated plant interactions with aboveground herbivores are not that striking compared to well-known interactions with soil antagonists (e.g. pathogens), perhaps because the plant root system is a more direct interface for interactions between mycorrhizal fungi and belowground antagonists [8]. Together, our perspectives involving phytochemicals and mycorrhizal associations will inspire future studies to focus on more accurate species-level data to understand their roles in modifying plant-herbivore interactions and consequently ecosystem functions at a macroecological scale.

## Supplementary Material

nwad236_Supplemental_Files
