# Supplementary material for: Global latitudinal patterns in leaf herbivory are related to variation in climate, rather than phytochemicals or mycorrhizal types
Source: Natl Sci Rev. 2023 Sep 8;10(12):nwad236. doi: 10.1093/nsr/nwad236 (PMC10632796; doi:10.1093/nsr/nwad236)
Supplement: nwad236_Supplemental_Files [file nwad236_supplemental_files.zip › Tang et al 2023_supplementary data.docx]

**Supplementary data for the manuscript “Global latitudinal patterns in leaf herbivory are related to variation in climate, rather than phytochemicals or mycorrhizal types”**

Hui Tang^1^, Xianhui Zhu^1^, Yonglin Zhong^2^, Yuanzhi Li^1^, Wenqi Luo^1^, Hanlun Liu^1^, Patrice Descombes^3,4^, Alan C. Gange^5^, Chengjin Chu^1*^

^1^State Key Laboratory of Biocontrol, School of Ecology / School of Life Sciences, Sun Yat-sen University, 510275 Guangzhou, China

^2^Guangdong Provincial Key Laboratory of Silviculture, Protection and Utilization, Guangdong Academy of Forestry, 510520 Guangzhou, China

^3^Musée et Jardins botaniques cantonaux, Av. de Cour 14B, 1007 Lausanne, Switzerland

^4^Department of Ecology and Evolution, University of Lausanne, Biophore, 1015 Lausanne, Switzerland

^5^Department of Biological Sciences, Royal Holloway University of London, Egham, Surrey TW20 0EX, UK

**^*^Corresponding author:** Chengjin Chu, [chuchjin@mail.sysu.edu.cn](mailto:chuchjin@mail.sysu.edu.cn)

**1.** **SUPPLEMENTARY MATERIALS AND METHODS**

**1.1 Literature search and herbivory data collection**

In this study, we compiled leaf herbivory data from three sources. First, we obtained leaf herbivory data from a previous study (1) which was based on two published datasets (2, 3) and other literature searched online by the end of 30 November 2014. Second, we searched the Web of Science for related literature published from 1 December 2014 to 27 August 2020 to expand the dataset. Third, we included data from a recent study (4), which reported leaf herbivory rates of 209 plant species from 65 families across continents. The current study was done to provide a better, more even global coverage across different biomes (5).

Specifically, we used the keywords ‘herbivory’, ‘leaf area loss’, ‘leaf damage’, ‘defoliation’, ‘folivory’ and ‘plant-herbivory interaction’ to search literature as used in previous studies (1). Studies were included for analyses only when they met the following criteria: (i) herbivory was measured as the proportion of leaf area consumed by all herbivores (e.g., leaf chewers, gallers and miners), in order to be comparable with existing datasets (1-3); (ii) we only included studies capable of capturing most of the damage during the lifetime of a leaf, e.g., data that quantified accumulated damage present on mature leaves, data collected at the end of the growing season and accumulated damage of individual leaves over one month or more were included (3); (iii) in the case of experimental studies, only data from control treatments were considered; and (iv) only data collected in field settings were considered. If herbivory was measured as a time series, the mean value of various time points was used. Means, standard errors/deviations and sample sizes were extracted from texts, tables or figures (1). For data presented in figures, we used the GetData image tool to extract exact values. Information on the latitude and longitude of the study sites and the growth form of the study species was collected. For target species that had multiple herbivory values recorded at a given location, and for some sites may be included in repeated studies, we combined the whole dataset to calculate mean herbivory values per plant species per location.

**1.2 Climate and elevation data collection**

We collected the mean annual temperature (MAT), mean annual precipitation (MAP) and elevation from original literature. For studies where climatic variables and elevation were not reported, we extracted them from WorldClim 2.1 climatic dataset with 30-second spatial resolution (6). We also extracted other 17 climatic variables along with solar radiation (SRAD) from WorldClim 2.1 (6) and extracted aridity index (AI) and potential evapotranspiration (PET) from the Global Aridity Index and Potential Evapotranspiration Database-Version 3 (7).

**1.3 Phytochemical data and mycorrhizal types**

We obtained phytochemical data from a recent synthesized study of phytochemicals across seed plants (8), which comprehensively compiled data on phytochemical presence from journals, books and a chemistry database (SciFinder: <https://scifinder.cas.org/>). According to their biosynthetic pathways, phytochemicals were divided into eight broad chemical classes: alkaloids, phenolic acids, flavonoids, tannins, terpenoids, steroids, quinones and phenylpropanoids (8). In this dataset, phytochemical data were qualitatively measured as presence (coded as 1) and absence or unclear (coded as 0) in all known taxa within the family for a certain phytochemical class. Considering the multidimensional nature of phytochemical diversity and availability of a phytochemical dataset (8), here we defined the phytochemical diversity as the total number of phytochemical classes within each plant family (9, 10). Although phytochemical information was initially collected at the plant species or genus level, sampled species for phytochemicals do not exactly match the species for leaf herbivory. We then assigned the number of chemical classes in a plant family to each species within the family, given that closely related plant species often possess similar secondary metabolites in traditional compound classes (11). In our dataset, phytochemical diversity ranged from zero to eight (Supplementary Fig. S4). Since the source dataset did not include all potential phytochemicals (8), the plant families with zero classes of phytochemicals may contain compounds that cannot be captured by traditional analytical methods like liquid chromatography and mass spectrometry (LC-MS). Therefore, we should interpret the zero classes of phytochemicals as that we do not find phytochemicals belonging to the above eight broad classes based on currently available chemical data and analytical approaches.

Each plant species was initially assigned to one of the four mycorrhizal types: AM, EcM, NM or other mycorrhizal types, following FungalRoot, a global database of plant mycorrhizal associations (12, 13). All mycorrhizal types other than AM, EcM or NM were combined as ‘Others’, since the three types, AM, EcM or NM, are linked to our scientific questions and most common in our dataset. AM-associated species, EcM-associated species, nonmycorrhizal plants and a clumped category for all other mycorrhizal types were then simplified as AM plants, EcM plants, NM plants and ‘Others’ plants, respectively. The basic flowchart was that we assigned mycorrhizal type at the plant genus level as suggested (12, 13).

**1.4 Statistical analyses**

Herbivory data are continuous proportions and can be modelled by a beta probability distribution which constrains values in an open interval (0,1) (14). To deal with the 0 values in our dataset, for all data points we used a linear transformation y*= (y(n−1)+0.5)/n, where n is the total number of observations (14). This approach models proportional data at their original scale, which makes it more straightforward for statistical inference and less biased for parameter estimates compared to traditional nonlinear transformations (e.g., logit or arcsine). Since closely related plant species are expected to have similar herbivory levels, we needed to account for phylogenetic relationships among plant species in models. We generated the phylogenetic tree of all plant species in our dataset using the ‘V.PhyloMaker’ package which contains a huge dated backbone phylogenetic tree for vascular plants (15).

In this study we conducted the following three interconnected sections of statistical analyses. First, we used a phylogenetic generalized linear mixed model (PGLMM) to test the global latitudinal patterns of leaf herbivory with a beta error family and logit link function in the ‘phyloglmm’ package (16). Considering potentially nonlinear latitudinal trend and the elevation effects on leaf herbivory, we modelled herbivory rates as a function of latitude, its quadratic term, elevation and elevation by latitude interaction term, with plant species, location and data source included as random intercept effects. We used the ΔAIC to represent the difference of a quadratic model AIC value minus that of a linear model without the quadratic latitude term, and a ΔAIC less than -2 indicates the quadratic model was the better one. Marginal R-squared values ($R_{m}^{2}$) and conditional R-squared values ($R_{c}^{2}$) of models were obtained in the ‘performance’ package (17). To further accounting for the elevation effect on latitudinal pattern in herbivory, we also applied alternative approach by using elevation-weighted latitudes. Generally, a 100-m upward shift is thermally equivalent to a 100-km poleward shift, and one degree of latitude is equivalent to 111 km of geographic distance (18). Therefore, we used the following formula to obtain elevation-adjusted latitude (18-19):

Adjusted latitude = elevation/111 + |latitude|

To resolve the potential problem of uneven sampling across latitudes, we evened out sample size across latitudes by randomly resampling one data point (herbivory rate of one species) for each location, considering that some locations had sampled only one species. We conducted the random resample process 1000 times and ran the PGLMM analysis above for each of the 1000 subsamples.

Second, we explored the relative effects of various abiotic and biotic factors on herbivory rates using the similar PGLMM analysis mentioned above. Specifically, we included abiotic factors (climatic variables and their quadratic terms) and biotic factors (phytochemical diversity, plant growth form and mycorrhizal type) as fixed effects, with plant species, location and data source included as random intercept effects. Considering potential nonlinear climate effects on insect herbivory, we thus added quadratic terms of climatic variables in above models. Many of the total 22 climatic variables were highly correlated with one another, and MAT and MAP were suggested to be the two most dominant climatic variables in ecology. We then constructed a correlation matrix for these 22 climatic variables and selected those whose correlation coefficients with MAT, MAP or each other were less than 0.6 to avoid multicollinearity in model fitting. Some of these selected variables were highly correlated with each other and had a correlation coefficient no less than 0.6. Here we also used the ΔAIC metric above to determine which climatic variables and/or their quadratic terms should retain in the final model. All continuous climatic variables were scaled to have means of 0 and standard deviations of 1 before model fitting so that their effects can be comparable. The growth form (woody vs non-woody) was showed to be a predictor of herbivory (1), and was also included in our model. Among the variables included in models, herbivory, latitude, MAT and MAP are continuous quantitative variables, phytochemical diversity is a discrete quantitative variable, and growth form and mycorrhizal type are qualitative or categorical variables. For plant mycorrhizal type, only AM, EcM and NM plants are closely related to our research, so for the sake of conciseness the ‘Others’ group was included for statistical analyses but not shown in visualization.

Third, after exploring effects of climatic variables, phytochemical diversity and plant mycorrhizal type on herbivory, we further reveal latitudinal variations of these variables in order to better understand how they influence global latitudinal patterns in herbivory. We used the flexible generalized additive models (GAM) to examine potentially nonlinear climate-latitude relationships separately for each climatic variable in the ‘mgcv’ package (20). In order to test whether phytochemical diversity varied with latitude, we performed a PGLMM analysis. This PGLMM model treated number of classes of phytochemicals as response, latitude as a fixed effect, and plant species and location as random intercept effects, with a poisson error family and a log link function. Then, we compared the latitudinal distribution of different plant mycorrhizal types by conducting a nonparametric Kruskal-Wallis test due to the violation of data normality, followed by a Dunn's test of multiple comparisons. All statistical analyses were performed on the R platform version 4.3.1 (21).

**2. SUPPLEMENTARY RESULTS**


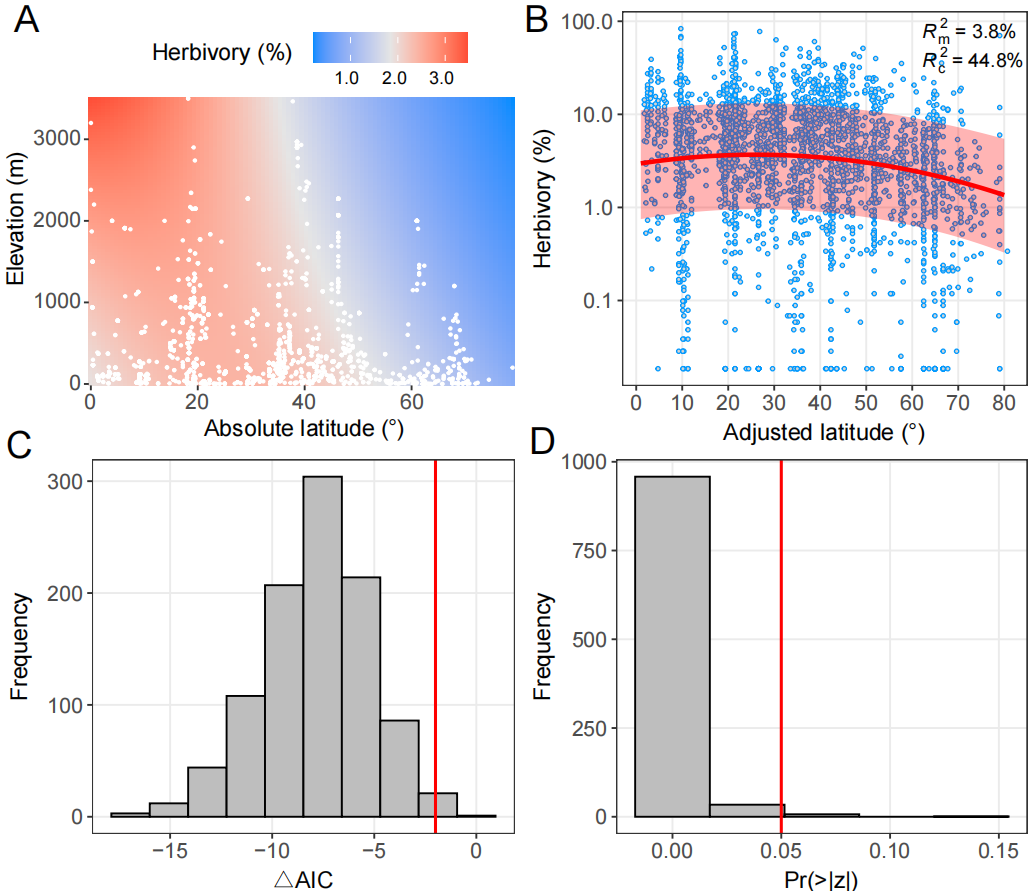


**Supplementary Figure S1** The PGLMM analyses for latitudinal patterns in herbivory when accounting for the elevation effect and sampling effect. (A) The effect of absolute latitude by elevation interaction on herbivory. White scatter points on the heat plot were sampling locations. (B) The global herbivory pattern along absolute latitudes adjusted by elevation. Shown are the marginal R-squared value ($R_{m}^{2}$) which represents the proportion of the variance explained by fixed effects, and the conditional R-squared value ($R_{c}^{2}$) which represents the proportion of the variation explained by both fixed and random effects. (C) Comparison between the quadratic and linear models for 1000 randomly resampled subsamples. ΔAIC was the difference between AIC values of the two models; ΔAIC less than -2 indicates the quadratic model was the better one. The vertical red line indicated the ΔAIC value of -2. (D) Statistical significance of the quadratic term of latitude in quadratic models in the panel C. The red vertical line indicated 0.05 value of Pr(|z|).


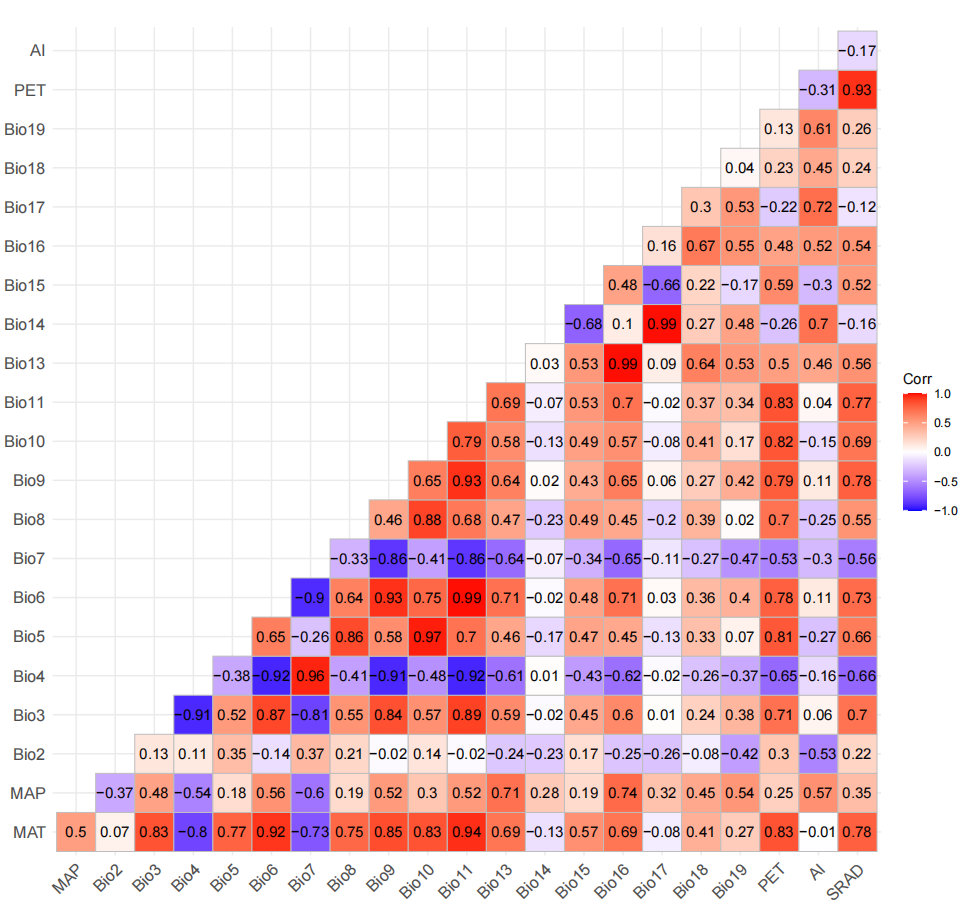


**Supplementary Figure S2** The correlation matrix of 22 climate variables for sampling locations. Abbreviations: MAT, mean annual temperature; MAT, mean annual precipitation; Bio2, mean diurnal range; Bio3, isothermality; Bio4, temperature seasonality; Bio5, max temperature of warmest month; Bio6, min temperature of coldest month; Bio7, temperature annual range; Bio8, mean temperature of wettest quarter; Bio9, mean temperature of driest quarter; Bio10, mean temperature of warmest quarter; Bio11, mean temperature of coldest quarter; Bio13, precipitation of wettest month; Bio14, precipitation of driest month; Bio15, precipitation seasonality; Bio16, precipitation of wettest quarter; Bio17, precipitation of driest quarter; Bio18, precipitation of warmest quarter; Bio19, precipitation of coldest quarter; PET, potential evapotranspiration; AI, aridity index; SRAD, solar radiation.


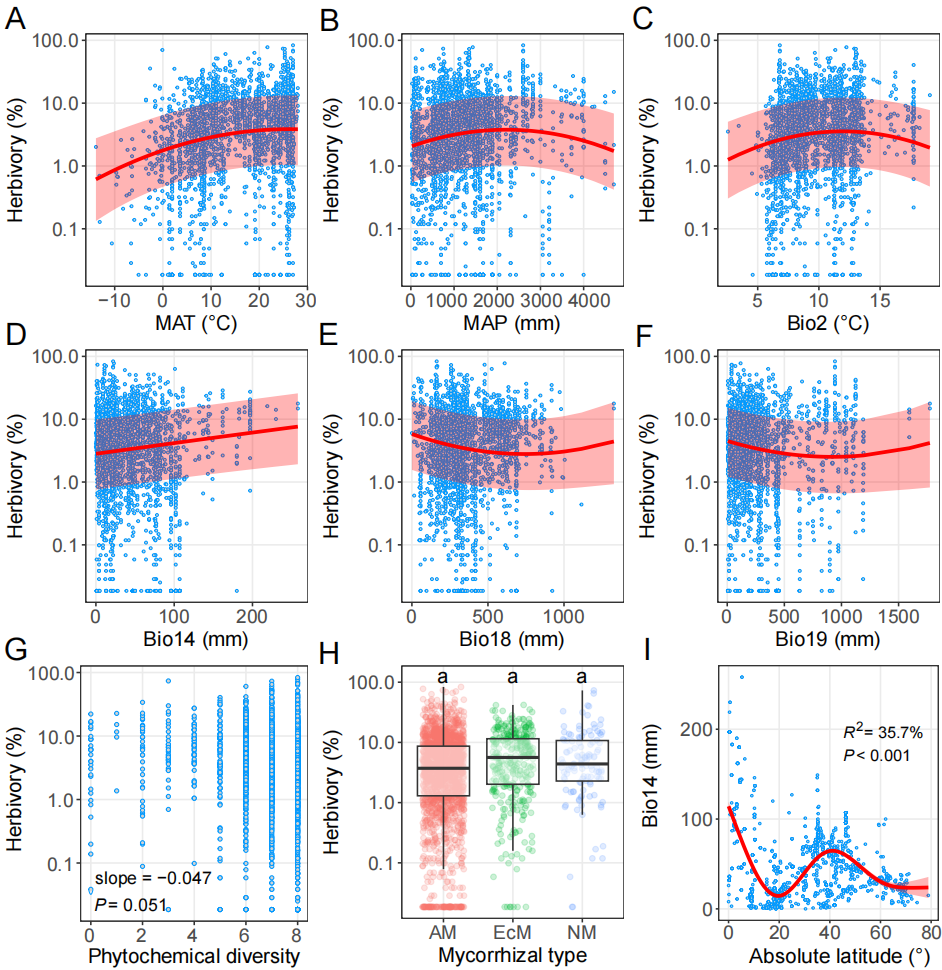


**Supplementary Figure S3** Main results of the best PGLMM Model 2 in the Supplementary Table S2. The abbreviation of climatic variables followed the Supplementary Figure S2. More detailed statistical results were in the Supplementary Table S4


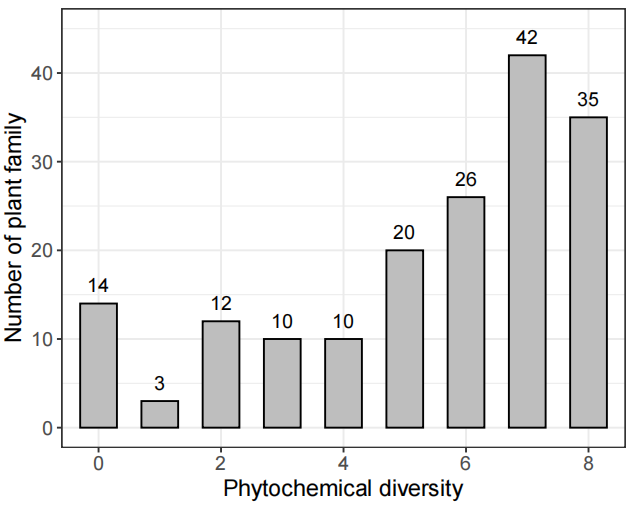


**Supplementary Figure S4** The distribution of phytochemical diversity in plant families where the herbivory data were collected.


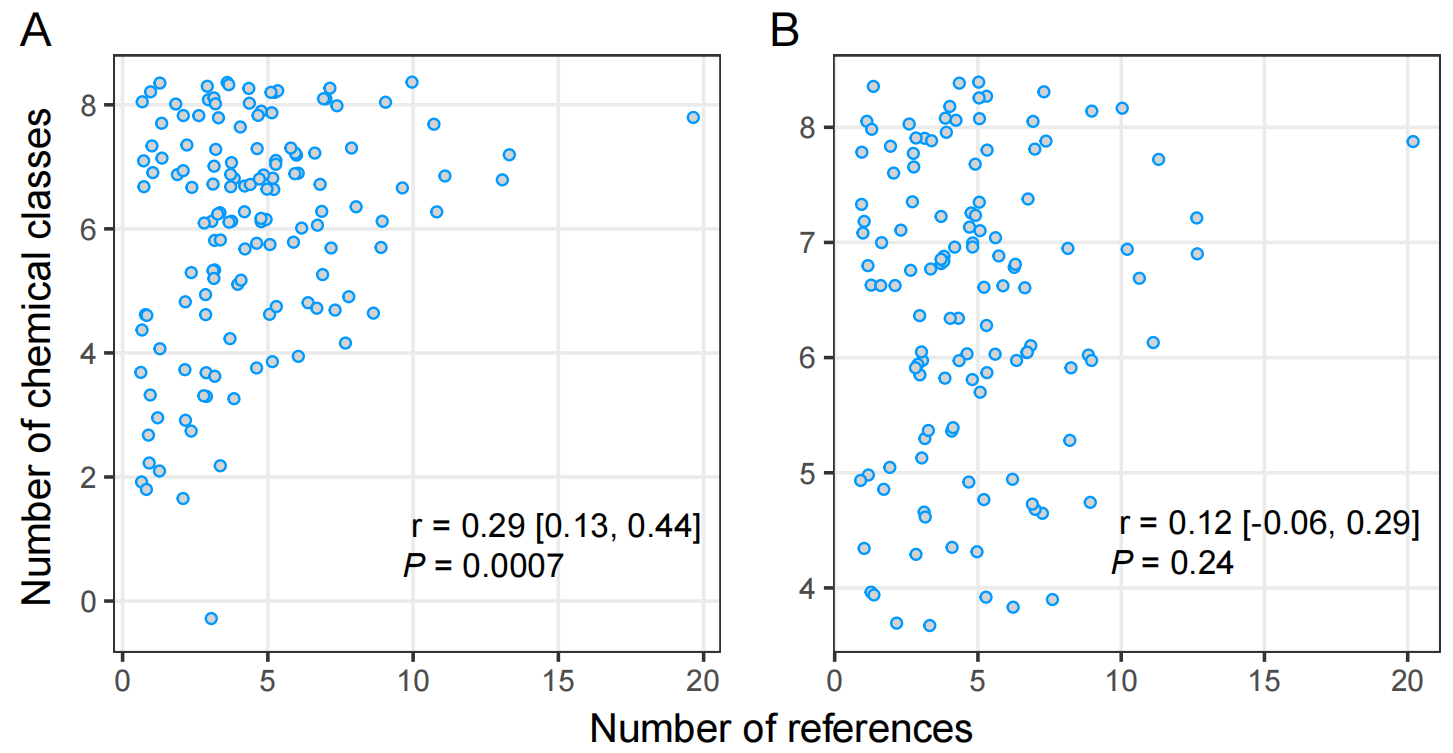


**Supplementary Figure S5** Correlation between the number of reference and the number of chemical class for each plant family according to a previous study (8). (A) Correlation analysis for all plant families; (B) Correlation analysis for plant families that had more than three classes of phytochemicals to be found. Shown were Pearson’s r correlation coefficients and their 95% confidence intervals. Scatter points were slightly jittered for better visualization.


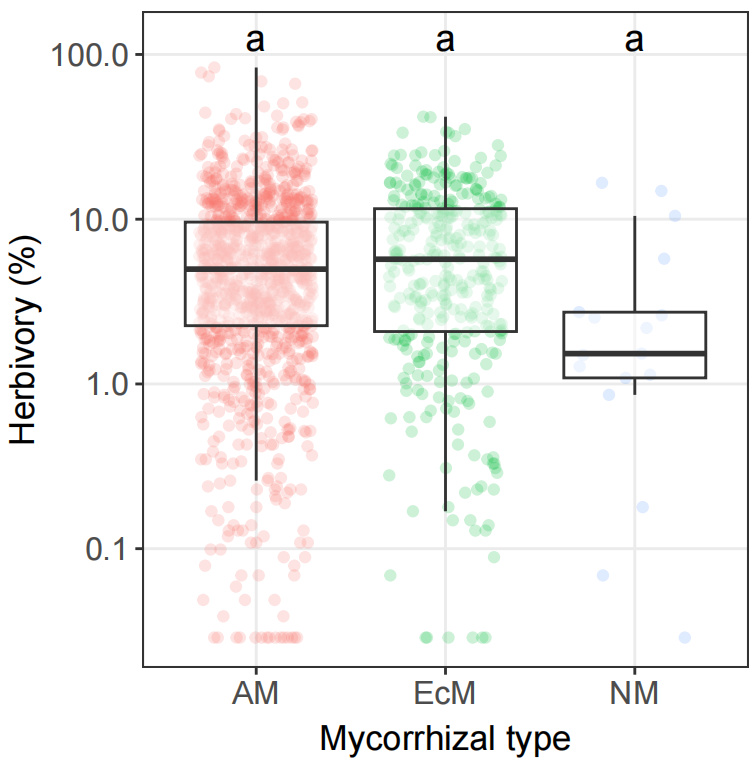


**Supplementary Figure S6** The marginal effect of mycorrhizal type on herbivory for woody species when accounting for the effects of climate and phytochemical diversity. Shown were plants associated with different mycorrhizal types: arbuscular mycorrhizal (AM), ectomycorrhizal (EcM), nonmycorrhizal (NM). Letters above the boxplots indicate groups identified by the Tukey's HSD multiple comparison analyses.

**Supplementary Table S1.** Latitude-herbivory relationships were tested using the phylogenetic generalized linear mixed model (PGLMM). Plant species, location and data source were included as random intercept effects. Abbreviations: Latitude^2^, the quadratic term of latitude; SD, standard deviation. ΔAIC, the difference of a quadratic model AIC value minus that of a linear model without the quadratic latitude term, was -10.47 (-10619.29 VS -10608.82). For all tables: *, *P* < 0.05; **, *P* < 0.01; ***, *P* < 0.001.

| **Effect** | **Term** | **Estimate** | **Std. error** | ***z* value** | ***P-*value** |
| --- | --- | --- | --- | --- | --- |
| Fixed | Intercept | -3.526 | 0.716 | -4.924 | **8.46e-07 ***** |
|  | Latitude | 0.020 | 0.008 | 2.538 | **0.011*** |
|  | Latitude^2^ | -0.0004 | 0.0001 | -3.543 | **0.0004 ***** |
|  | Elevation | 0.0002 | 0.0001 | 1.576 | 0.115 |
|  | Latitude:Elevation | -7.073e-06 | 3.321e-06 | -2.130 | **0.033*** |
| Random | SD of (1\|Species) | 0.065 |  |  |  |
|  | SD of (1\|Location) | 0.642 |  |  |  |
|  | SD of (1\|Source) | 0.154 |  |  |  |
| R^2^ | Marginal R^2^ | 0.039 |  |  |  |
|  | Conditional R^2^ | 0.449 |  |  |  |

**Supplementary Table S2. Selection of climatic variables in the PGLMM models.** Here, the quadratic model included all the climatic variables listed and their quadratic terms, while the linear model only included climatic variables themselves. Except for this, the quadratic and linear model had the same set of biotic variables (phytochemical diversity, plant mycorrhizal type and growth form) and random effects (plant species, location and data source). The abbreviation of climatic variables followed Supplementary Figure S2. The best models with the lowest AIC values were marked in bold. Since the two best models had similar results, we reported the results of the Model 1 in the main text and the results of the Model 2 in the Supplementary materials.

| **Model** | **Code** | **Climatic variable** | **AIC value** |
| --- | --- | --- | --- |
| **Quadratic**  **model** | **Model 1**  **Model 2**  Model 3  Model 4 | **MAT + MAP + Bio2 + Bio15 + Bio18 + Bio19**  **MAT + MAP + Bio2 + Bio14 + Bio18 + Bio19**  MAT + MAP + Bio2 + Bio14 + Bio18 + AI  MAT + MAP + Bio2 + Bio17 + Bio18 | **-10679. 86**  **-10678.56**  -10672.05  -10669.84 |
| Linear model | Model 5  Model 6  Model 7  Model 8 | MAT + MAP + Bio2 + Bio15 + Bio18 + Bio19  MAT + MAP + Bio2 + Bio14 + Bio18 + Bio19  MAT + MAP + Bio2 + Bio14 + Bio18 + AI  MAT + MAP + Bio2 + Bio17 + Bio18 | -10641.17  -10640.91  -10640.61  -10636.85 |

**Supplementary Table S3.** Summary results of the best PGLMM Model 1 in the Supplementary Table S2 that tested the effects of abiotic and biotic factors on herbivory. Plant species, location and data source were included as random intercept effects. All climatic variables were scaled to had means of 0 and standard deviations of 1 before model fitting. Variable abbreviations: MAT, mean annual temperature; MAP, mean annual precipitation; MAP^2^, the quadratic term of MAP; Bio2, mean diurnal range; Bio15, precipitation seasonality; Bio18, precipitation of warmest quarter; Bio19, precipitation of coldest quarter; PD, phytochemical diversity; MycorrType, plant mycorrhizal type; SD, standard deviation. The quadratic terms, MAT^2^ and (Bio15)^2^, were not statistically significant, so they were removed from the final model for better parameter estimate. For all tables: *, *P* < 0.05; **, *P* < 0.01; ***, *P* < 0.001.

| **Effect** | **Term** | **Estimate** | **Std. error** | ***z* value** | ***P-*value** |
| --- | --- | --- | --- | --- | --- |
| Fixed | Intercept | -3.221 | 0.718 | -4.490 | **<0.001***** |
|  | MAT | 0.301 | 0.055 | 5.478 | **<0.001***** |
|  | MAP | 0.217 | 0.051 | 4.280 | **<0.001***** |
|  | Bio2 | 0.190 | 0.044 | 4.342 | **<0.001***** |
|  | Bio15 | -0.194 | 0.048 | -4.021 | **<0.001***** |
|  | Bio18 | -0.141 | 0.042 | -3.388 | **<0.001***** |
|  | Bio19 | -0.223 | 0.066 | -3.396 | **<0.001***** |
|  | MAP^2^ | -0.091 | 0.018 | -4.985 | **<0.001***** |
|  | (Bio2)^2^ | -0.087 | 0.026 | -3.347 | **<0.001***** |
|  | (Bio18)^2^ | 0.058 | 0.021 | 2.817 | **0.005**** |
|  | (Bio19)^2^ | 0.052 | 0.022 | 2.317 | **0.021*** |
|  | PD | -0.048 | 0.024 | -1.992 | **0.046*** |
|  | Growth form [Woody] | 0.160 | 0.081 | 1.952 | 0.051 |
|  | MycorrType [AM] | -0.005 | 0.211 | -0.023 | 0.982 |
|  | MycorrType [EcM] | 0.136 | 0.275 | 0.496 | 0.620 |
|  | MycorrType [Others] | 0.070 | 0.214 | 0.328 | 0.743 |
| Random | SD of (1\|Species) | 0.063 |  |  |  |
|  | SD of (1\|Location) | 0.608 |  |  |  |
|  | SD of (1\|Source) | 0.206 |  |  |  |
| R^2^ | Marginal R^2^ | 0.121 |  |  |  |
| AIC | Conditional R^2^  -10680.33 | 0.483 |  |  |  |

**Supplementary Table S4** Summary results of the best PGLMM Model 2 in the Supplementary Table S2 that tested the effects of abiotic and biotic factors on herbivory. Plant species, location and data source were included as random intercept effects. All climatic variables were scaled to had means of 0 and standard deviations of 1 before model fitting. The abbreviation of climatic variables followed the Supplementary Table S3 and Supplementary Figure S2. The quadratic terms (Bio14)^2^, were not statistically significant, so it was removed from the final model for better parameter estimate. For all tables: *, *P* < 0.05; **, *P* < 0.01; ***, *P* < 0.001.

| **Effect** | **Term** | **Estimate** | **Std. error** | ***z* value** | ***P-*value** |
| --- | --- | --- | --- | --- | --- |
| Fixed | Intercept | -3.170 | 0.719 | -4.407 | **<0.001***** |
|  | MAT | 0.225 | 0.054 | 4.184 | **<0.001***** |
|  | MAP | 0.199 | 0.050 | 3.984 | **<0.001***** |
|  | Bio2 | 0.137 | 0.045 | 3.056 | **<0.001***** |
|  | Bio14 | 0.156 | 0.050 | 3.116 | **0.002**** |
|  | Bio18 | -0.197 | 0.047 | -4.145 | **<0.001***** |
|  | Bio19 | -0.245 | 0.071 | -3.457 | **<0.001***** |
|  | MAT^2^ | -0.091 | 0.038 | -2.398 | **0.017*** |
|  | MAP^2^ | -0.084 | 0.018 | -4.634 | **<0.001***** |
|  | (Bio2)^2^ | -0.084 | 0.026 | -3.248 | **0.001**** |
|  | (Bio18)^2^ | 0.050 | 0.020 | 2.438 | **0.015*** |
|  | (Bio19)^2^ | 0.050 | 0.022 | 2.259 | **0.024*** |
|  | PD | -0.047 | 0.024 | -1.948 | 0.051 |
|  | Growth form [Woody] | 0.159 | 0.081 | 1.952 | 0.051 |
|  | MycorrType [AM] | -0.013 | 0.211 | -0.060 | 0.952 |
|  | MycorrType [EcM] | 0.148 | 0.275 | 0.539 | 0.590 |
|  | MycorrType [Others] | 0.088 | 0.214 | 0.412 | 0.681 |
| Random | SD of (1\|Species) | 0.063 |  |  |  |
|  | SD of (1\|Location) | 0.608 |  |  |  |
|  | SD of (1\|Source) | 0.216 |  |  |  |
| R^2^ | Marginal R^2^ | 0.122 |  |  |  |
| AIC | Conditional R^2^  -10679.29 | 0.486 |  |  |  |

**REFERENCES**

1. Zhang, S, Zhang, Y, Ma, K. Latitudinal variation in herbivory: hemispheric asymmetries and the role of climatic drivers. *J Ecol*. 2016; **104**(4): 1089-95.

2. Kozlov, MV, Zverev, V, Zvereva, EL. Confirmation bias leads to overestimation of losses of woody plant foliage to insect herbivores in tropical regions. *PeerJ*. 2014; **2**(12): e709.

3. Turcotte, MM, Thomsen, CJM, Broadhead, GT*, et al.* Percentage leaf herbivory across vascular plant species. *Ecology*. 2014; **95**(3): 788-.

4. Mendes, GM, Silveira, FAO, Oliveira, C*, et al.* How much leaf area do insects eat? A data set of insect herbivory sampled globally with a standardized protocol. *Ecology*. 2021; **102**(4): e03301.

5. Whittaker RH. *Communities and Ecosystems*. Macmillan Publishing.1975.

6. Fick, SE. and RJ. Hijmans. WorldClim 2: new 1km spatial resolution climate surfaces for global land areas. *Int. J. Climatol.* 2017; **37**(12): 4302-4315

7. Zomer, RJ., Xu, J. & Trabucco, A. Version 3 of the Global Aridity Index and Potential Evapotranspiration Database. *Sci Data*; 2022; **9**, 409.

8. Zhang, Y, Deng, T, Sun, L*, et al.* Phylogenetic patterns suggest frequent multiple origins of secondary metabolites across the seed-plant ‘tree of life’. *Natl. Sci. Rev.* 2020; **8**(4): nwaa105.

9. Kessler, A, Kalske, A. Plant secondary metabolite diversity and species interactions. *Annu. Rev. Ecol. Evol. Syst*. 2018; **49**: 115-38.

10. Wetzel, WC, Whitehead, SR. The many dimensions of phytochemical diversity: Linking theory to practice. *Ecol Lett*. 2020; **23**(1): 16-32.

11. Zhu, F, Qin, C, Tao, L*, et al.* Clustered patterns of species origins of nature-derived drugs and clues for future bioprospecting. *Proc. Natl. Acad. Sci. U. S. A.* 2011; **108**(31): 12943-8.

12. Soudzilovskaia, NA, Vaessen, S, Barcelo, M*, et al.* FungalRoot: global online database of plant mycorrhizal associations. *New Phytol*. 2020; **227**(3): 955-66.

13. Soudzilovskaia, NA, He, J, Rahimlou, S*, et al.* FungalRoot v.2.0 – an empirical database of plant mycorrhizal traits. *New Phytol*. 2022; **235**(5): 1689-91.

14. Douma, JC, Weedon, JT. Analysing continuous proportions in ecology and evolution: A practical introduction to beta and Dirichlet regression. *Methods Ecol. Evol*. 2019; **10**(9): 1412-30.

15. Jin, Y, Qian, H. V.PhyloMaker: an R package that can generate very large phylogenies for vascular plants. *Ecography*. 2019; **42**(8): 1353-9.

16. Li, M, Bolker, B. phyloglmm: Machinery for phylogenetic GLMMs. R package version 0.1.0.9001. 2022. https://github.com/wzmli/phyloglmm.

17. Lüdecke, D, Ben-Shachar, MS, Patil, I*, et al.* performance: An R package for assessment, comparison and testing of statistical models. *Journal of Open Source Software*. 2021; **6**(60): 3139.

18. Jump, A. S., Matyas, C. & Penuelas, J. The altitude-for-latitude disparity in the

range retractions of woody species. *Trends Ecol. Evol*. 2009; **24**, 694–701

19. Zhong, Y, Chu, C, Myers, JA*, et al.* Arbuscular mycorrhizal trees influence the latitudinal beta-diversity gradient of tree communities in forests worldwide. *Nat. Commun*. 2021; **12**(1): 3137.

20. Wood SN, Augustin NH. GAMs with integrated model selection using penalized regression splines and applications to environmental modelling. *Ecol. Model.* 2002;**157**:157–177.

21. R Core Team. R: A language and environment for statistical computing. R foundation for statistical computing, Vienna, Austria. https://www.R-project.org/. 2023.
